# Inhibition of apoptosis in human tumour cells by the tumour-associated serpin, SCC antigen-1

**DOI:** 10.1054/bjoc.1999.1028

**Published:** 2000-02

**Authors:** Y Suminami, S Nagashima, N L Vujanovic, K Hirabayashi, H Kato, T L Whiteside

**Affiliations:** University of Pittsburgh Cancer Institute, Department of Obstetrics and Gynecology, Yamaguchi University School of Medicine, Ube, 755-8505, Japan; Department of Pathology and Otolaryngology, University of Pittsburgh School of Medicine, 200 Lothrop Street, W1041 BST, Pittsburgh, PA, 15213, USA

**Keywords:** tumour-associated protein, serpin, SCC Ag-1, apoptosis, protection from apoptosis

## Abstract

The squamous cell carcinoma antigen (SCC Ag) is a tumour-associated protein and a member of theserineproteaseinhibitor (serpin) family. The SCC Ag has been used as a serologic tumour marker for SCC progression, and its elevated serum levels are a risk factor for disease relapse. However, the biologic significance of this intracytoplasmic protein in cancer cells remains unknown. In this report, we demonstrated that apoptosis induced by 7-ethyl-10-hydroxycamptothecin, tumour necrosis factor-α (TNF-α) or interleukin (IL)-2-activated natural killer (NK) cells was significantly inhibited in tumour cells transduced with the *SCC Ag-1* cDNA, as compared to control cells in vitro. Also, inhibition of the SCC Ag-1 expression in tumour cells by transfection of antisense *SCC Ag-1* cDNA was accompanied by significantly increased sensitivity of these cells to apoptosis induced by etoposide or TNF-α. The mechanism of protection of tumour cells from apoptosis involved inhibition of caspase-3 activity and/or upstream proteases. In vivo, tumour cells overexpressing the SCC Ag-1 formed significantly larger tumours in nude mice than the SCC Ag-1-negative controls. Thus, overexpression of the SCC Ag-1, a member of the serpin family, in human cancer cells contributed to their survival by mediating protection from drug-, cytokine- or effector cell-induced apoptosis. © 2000 Cancer Research Campaign


*British Journal of Cancer* (2000) **82**(4), 981–989
© 2000 Cancer Research Campaign
DOI: 10.1054/ bjoc.1999.1028, available online at http://www.idealibrary.com on IDEAL®

### Inhibition of apoptosis in human tumour cells by the tumour-associated serpin, SCC antigen-1

**Y Suminami[1,2], S Nagashima[1], NL Vujanovic[1], K Hirabayashi[2], H Kato[2] and TL Whiteside[1,3]**

[1]University of Pittsburgh Cancer Institute and [3]Department of Pathology and Otolaryngology, University of Pittsburgh School of Medicine, 200 Lothrop Street, W1041 BST, Pittsburgh, PA 15213, USA; [2]Department of Obstetrics and Gynecology, Yamaguchi University School of Medicine, Ube 755-8505, Japan

**Summary** The squamous cell carcinoma antigen (SCC Ag) is a tumour-associated protein and a member of the serine protease inhibitor (serpin) family. The SCC Ag has been used as a serologic tumour marker for SCC progression, and its elevated serum levels are a risk factor for disease relapse. However, the biologic significance of this intracytoplasmic protein in cancer cells remains unknown. In this report, we demonstrated that apoptosis induced by 7-ethyl-10-hydroxycamptothecin, tumour necrosis factor-α (TNF-α) or interleukin (IL)-2-activated natural killer (NK) cells was significantly inhibited in tumour cells transduced with the *SCC Ag-1* cDNA, as compared to control cells in vitro. Also, inhibition of the SCC Ag-1 expression in tumour cells by transfection of antisense *SCC Ag-1* cDNA was accompanied by significantly increased sensitivity of these cells to apoptosis induced by etoposide or TNF-α. The mechanism of protection of tumour cells from apoptosis involved inhibition of caspase-3 activity and/or upstream proteases. In vivo, tumour cells overexpressing the SCC Ag-1 formed significantly larger tumours in nude mice than the SCC Ag-1-negative controls. Thus, overexpression of the SCC Ag-1, a member of the serpin family, in human cancer cells contributed to their survival by mediating protection from drug-, cytokine- or effector cell-induced apoptosis. © 2000 Cancer Research Campaign

**Keywords**: tumour-associated protein; serpin; SCC Ag-1; apoptosis; protection from apoptosis

The squamous cell carcinoma antigen (SCC Ag) was first discovered as a tumour-associated protein in squamous cell carcinoma of the uterine cervix and named the TA-4 (Kato and Torigoe, 1977). This protein was also found to be expressed in the normal squamous epithelium, but its level was found to be significantly increased in cancer tissues as well as sera of patients with SCC. Therefore, the SCC Ag has been used as a tumour marker for SCC of various organs, including uterine cervix, skin, head and neck, oesophagus, lung and bladder (Kato et al, 1979; Mino et al, 1988; Kato, 1992). In fact, elevated serum levels of the SCC Ag antigen have been shown to correlate with the clinical stage of SCC, ranging from 18% at stage 0 to > 90% at stage IV of uterine cervical SCC (Kato, 1992). After tumour resection, serum levels of the SCC Ag fall rapidly, and the subsequent increase of its level strongly indicates recurrence of the disease. Furthermore, at stages Ib and IIa of uterine cervical cancer, elevated serum levels of the SCC Ag prior to treatment are a risk factor for disease recurrence, independent of the tumour size or lymph node metastases (Duk et al, 1996). This tumour marker can be separated into two fractions (neutral and acidic) by isoelectric focusing (Kato et al, 1984), and recent studies indicate that there are two genes encoding SCC Ag (Schneider et al, 1995). They encode two proteins designated as the SCC Ag-1 and the SCC Ag-2, which have 92% homology.

Although the level of expression of the SCC Ag increases in tissue and serum of patients with cancer, biologic function of the SCC Ag in cancer cells has remained undefined. We have cloned cDNA of the SCC Ag-1 (Suminami et al, 1991) and identified it as a member of the ovalbumin-serine protease inhibitor (ov-serpin) family (Suminami et al, 1991; Potempa et al, 1994). Subsequently, it has been shown that the recombinant SCC Ag-1 can bind to and inhibit chymotrypsin (serine protease) (Nawata et al, 1995) and cathepsin L (cysteine protease) in vitro (Takeda et al, 1995; Nawata et al, 1997). Proteins of the ov-serpin family have unique characteristics, which distinguish them from other members of the serpin super family. While most serpins are secreted and work extracellulary, ov-serpins are intracellular protease inhibitors, which are secreted only occasionally and without a signal sequence by an unknown mechanism (Belin et al, 1989).

Various inhibitory mechanisms of apoptosis have been identified in cancer cells, which seem to augment the progression of cancer by interfering with death of cancer cells. For example, many cancer cells express the anti-apoptotic *bcl-2* gene (Reed, 1995). Nearly all anticancer drugs eliminate cancer cells by inducing apoptosis (Fisher, 1994; Ormerod et al, 1994; Desjardins and MacManus, 1995). Recent studies indicate that intracellular proteases are important mediators of apoptosis (Alnemri et al, 1996; Tewari et al, 1995*a*), and that protease inhibitors are involved in the regulation of cell death. Among the serpin family, a viral serpin (CrmA) and a human serpin (PAI-2) have been reported to inhibit apoptosis induced by cytolytic effector cells (CTL) and other stimuli (Tewari and Dixit, 1995; Tewari et al, 1995*b*) or by tumour necrosis factor-α (TNF-α) (Dickinson et al, 1995; Gan et al, 1995), respectively. However, these two serpins function as a part of inflammatory or infectious processes and, to the best of our knowledge, no report exists which indicates that the level of expression of serpins is related to tumour growth or that serpins have inhibitory effects on drug-induced apoptosis of tumour cells.

*Received 9 March 1999*
*Revised 29 July 1999*
*Accepted 5 August 1999*

*Correspondence to:* TL Whiteside

In this manuscript, we report that overexpression of the SCC Ag-1 attenuates apoptosis induced in human SCC cells by anti-cancer drugs, TNF-α or human interleukin (IL)-2-activated natural killer (NK) cells in vitro. Tumour cells overexpressing the SCC Ag-1 grow more rapidly in vivo in experimental animals than control cells. Our results suggest that the SCC Ag-1 has a stimulatory effect on progression of SCC due to its ability to attenuate tumour cell apoptosis, possibly at the level of caspase-3 or of the proteases operating upstream from caspase-3.

### MATERIALS AND METHODS

### Cell lines and reagents

PCI-51, a human head and neck squamous cell carcinoma (SCC) cell line, was established in our laboratory as previously described (Hirabayashi et al, 1995). K562, a human NK-sensitive cell line, derived from a patient with chronic myelogenous leukaemia, was maintained as described (Vujanovic et al, 1995*a*). Human A-NK cells were purified from peripheral blood mononuclear cells (PBMC) obtained from normal donors, using a negative selection method described previously (Vujanovic et al, 1993). A SCC cell line, SKG IIIa, used as a positive control, was a gift from Dr S Nozawa (Keio University, Japan). A mouse lung SCC cell line, KLN-205, used for in vivo experiments, was purchased from American Type Culture Collection (Rockville, MD, USA). Restriction enzymes and T4 DNA ligase were purchased from Boehringer Mannheim (Indianapolis, IN, USA). Recombinant human TNF-α, 7-ethyl-10-hydroxycamptothecin (SN-38) and etoposide were gifts from Knoll Pharmaceuticals (Whippany, NJ, USA), Daiichi Pharmaceutical Corp. (Tokyo, Japan) and Nippon Kayaku Corp. (Tokyo, Japan) respectively.

### Construction of the expression vector and transduction of the *SCC Ag-1* cDNA

The coding region of *SCC Ag-1* cDNA was amplified by polymerase chain reaction (PCR) from pKK-SCC-1 (Suminami et al, 1991) using the following primers: 5′-CAGACCATGGATTCACTCAGT-3′ (sense) and 5′-TCTGTTGTTGCCAGCAATCAG-3′ (antisense).

To create an *Nco*I site at the translation start codon for insertion into the MFG retroviral vector (Ohashi et al, 1992), a nucleotide was changed from A to G at position 65, which introduced Asp instead of Asn at the 2nd residue. PCR was performed with 30 cycles of 94°C, 55°C and 72°C (each for 1 min). The amplified fragment was cut completely with *Asp*700 and partially with *Nco*I. *Bam*HI adapter (Boehringer Mannheim, Indianapolis, IN, USA) was ligated to the blunt end of the fragment created by *Asp*700 and digested with *Bam*HI. This fragment was ligated to the *Nco*I and *Bam*HI cloning sites in the MFG retroviral vector. Next, the IRES-*neo*$^r$ cassette, containing *Bam*HI site on both ends (Zitvogel et al, 1994), was inserted into the *Bam*HI site of the MFG vector, and the direction of the insert was confirmed. This construct (MFG-SCC1-Neo), and the control construct without the SCC Ag-1 cDNA, were used for transfection of the retrovirus packaging cell line, CRIP (Danos and Mulligan, 1988) by the calcium–phosphate method. PCI-51, K562 and KLN-205 were incubated with supernatants of CRIP, infected and selected in G-418 medium (Gibco-BRL, Gaithersburg, MD, USA) in bulk cultures. The resulting transduced cells (PCI-51-SCC, K562-SCC and KLN-SCC) and control tumour cells transduced only with the neomycin resistance (*neo*$^r$) cDNA (PCI-51-NEO, K562-NEO and KLN-NEO) were used for the experiments described below. In the case of PCI-51, transfection was repeated to obtain tumour cells which expressed high levels of the SCC-Ag-1 (PCI-51 SCCre).

### Expression of the SCC Ag-1 cDNA in transduced cells

Semi-quantitative reverse transcription PCR (RT-PCR) was performed using a RNA-PCR kit (Perkin-Elmer, Norwalk, CT, USA) following the manufacturer's protocol with the SCC Ag-1 primer pair: 5′-CCAACAAGCTCTTCGGAGA-3′ (sense), 5′-TCTACGGGGATGAGAATCT-3′ (antisense) or the β-actin primer pair: 5′-GGGTCAGAAGGATTCCTATG-3′ (sense), 5′-GGTCTCAAACATGATCTGGG-3′ (antisense).

Reverse transcription was performed with 1 μg (SCC Ag-1) or 0.05 ng (β-actin) of total RNA at 42°C for 15 min. PCR amplification (35 cycles; confirmed in preliminary experiments to be in the exponential phase) was performed with denaturation at 94°C, annealing at 58°C and extension at 72°C (each for 1 min), adding 0.1 μl of [α-$^{32}$P] dCTP. Radioactivity of the amplicon band was counted in an image analyser (Phosphor Imager, Molecular Dynamics, Sunnyvale, CA, USA), and the ratio of the counts obtained with the SCC Ag-1 and β-actin was calculated. Western blotting was performed using the enhanced chemiluminescence (ECL) detection system (Amersham, Arlington Heights, IL, USA) with a monoclonal antibody (mAb-13) to SCC Ag-1 (Suminami et al, 1991). The expression level of the SCC Ag-1 protein was also analysed by a sensitive immunoassay method (IMx, Dainabot, Tokyo, Japan), as described previously (Takeshima et al, 1990).

Expression of TNF-α receptors (p55 and p75) was analysed by flow cytometry, using the receptor-specific mAbs and FACScan (Becton Dickinson, San Jose, CA, USA).

### Inhibition of SCC Ag-1 expression in the SCC cell line

RT-PCR was performed using total RNA from cervical cancer tissue and the following primers for SCC Ag-1: 5′-ACGAGGTACCTCACCATGAATTCACTCAG-3′ and 5′-AGCACTCGATCTACGGGGATGAGAATCT-3′ with 30 cycles at 94°C, 55°C, and 72°C (each for 1 min) followed by ligation with TA cloning vector (Invitrogen, Carlsbad, CA, USA). *Xho*I fragment of the insert was cut out and ligated with *Xho*I site of pCEP4 vector (Invitrogen, Carlsbad, CA, USA), and the antisense direction of the insert was confirmed. The antisense construct (pCEP4-SCCAS) or pCEP4 (control) was used to transduce SKG IIIa cells. After selection with hygromycin, the expression level of the SCC antigen in each clone was analysed in the IMx system.

### Apoptosis assays

Transduced cells were incubated with apoptosis-inducing drugs, SN-38 or etoposide (at the indicated concentrations), or TNF-α (10 ng ml$^{-1}$) after 4 h of preincubation with cycloheximide (10 ng ml$^{-1}$). Apoptotic tumour cells were identified morphologically after staining with Hoechst 33342 dye. Inhibition of apoptosis was determined by counting viable cells and expressed as the percentage of viability.

IL-2 activated NK cells (A-NK cells) used as effector cells were purified from PBMC obtained from normal donors and cultured as described previously (Vujanovic et al, 1993). Killing of transduced

 © 2000 Cancer Research Campaign

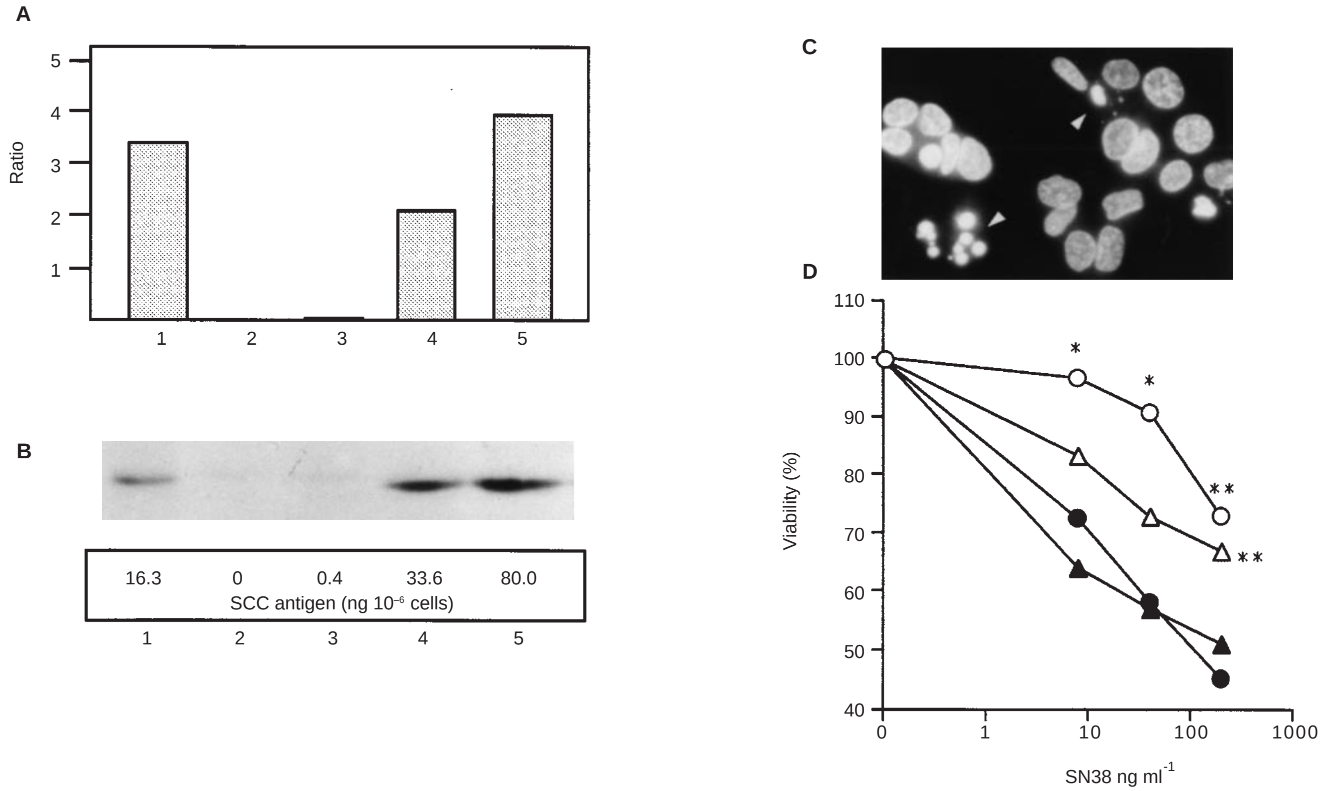

**Figure 1** Expression of the SCC Ag-1 in tumour cell lines transduced with the *SCC Ag-1* cDNA and its effect on SN-38-induced apoptosis. (**A**) Expression levels of *SCC Ag-1* mRNA were determined by semi-quantitative RT-PCR. The data shown are the ratio of cpm obtained in Phosphor Imager with the SCC Ag-1 and β-actin PCR bands. (**B**) The SCC Ag-1 protein was detected by Western blot analysis of cell lysates probed with anti-SCC Ag mAb. The level of the SCC Ag-1 in each lysate was quantitated by the IMx method and is given below each lane. SKGIIIa cells, which express the endogenous SCC Ag-1, were used as a positive control. Lane 1, SKG IIIa; lane 2, PCI-51; lane 3, PCI-51-NEO; lane 4, PCI-51-SCC; lane 5, PCI-51-SCCre. (**C**) Tumour cells (PCI-51-SCC) were treated with various concentrations of SN-38 for 24 h. Few apoptotic cells were observed after staining with the Hoechst 33342 dye (original magnification ×400). (**D**) The percentages of viable tumour cells after 24 h incubation with SN-38 are shown. Open circles, PCI-51-SCCre; open triangles, PCI-51-SCC; closed circles, PCI-51-NEO; closed triangles, PCI-51. The results are means of triplicate cultures. The standerd deviations were less than 10% of mean values. A representative experiment of three performed is shown. **P* < 0.05 compared to PCI-51 and PCI-51-NEO. ***P* < 0.05 compared to PCI-51

tumour cells by A-NK cells was analysed in 4 h $^{51}$Cr-release (CRC) and $^{3}$H-TdR-release (JAM) assays, which measure perforin-mediated membrane lysis and DNA fragmentation, respectively, as described by us previously (Vujanovic et al, 1996). An MTT (3-(4,5-dimethyl thiazol-2-yl)-2,5 diphenyl-tetrazolium bromide) assay was also performed, as previously described (Nagashima et al, 1997), to evaluate cell death attributable to both killing mechanisms. Each effector cell preparation was tested in the same assay against PCI targets transduced with the *SCC Ag-1* or with *neo*$^r$ cDNA. The percentage of cell death was determined for each assay as previously described (Nagashima et al, 1997), and lytic units (LU) were calculated, using a computer program based on the formula of Pross et al (1981). The percentages of suppression of cytotoxicity or apoptosis was calculated according to the following formula:

$$\% \text{ suppression} = (1\text{-Ls/Ln})\times 100$$

where Ls are LU of activity obtained with PCI-51-SCC targets, while Ln are LU of activity obtained with PCI-51-NEO (control) targets tested in the same assay. DNA fragmentation of transduced cells was also analysed by the terminal deoxynucleotidyl transferase-mediated dUTP-biotin nick end labelling (TUNEL) method, using reagents purchased from Boehringer Mannheim.

### Caspase-3 assay

Increases in caspase-3 activity were measured using ApoAlert CPP32 protease assay kit (Clontech, Palo Alto, CA, USA) according to the manufacturer's recommendations. After 4 h preincubation with cycloheximide (10 ng ml$^{-1}$), each of the transduced cell lines was incubated with or without TNF-α (10 ng ml$^{-1}$) in the presence of cycloheximide (10 ng ml$^{-1}$) for 15 h. The cells were harvested, counted, lysed with the lysis buffer and centrifuged. After incubation with the substrate (DEVD-pNA) for 1 h, the ratio of caspase-3 activity was obtained by measuring OD405 of TNF-α (+) cells and TNF-α (–) cells minus OD405 values for the cells incubated in the absence of the substrate.

### In vivo experiments with SCC Ag-1-transduced cells

A murine SCC cell line, KLN-205, able to grow in nude mice was used for in vivo experiments. Tumour cells transduced with the *SCC Ag-1* and *neo*$^r$ cDNAs (KLN-SCC) or *neo*$^r$ cDNA alone (KLN-NEO) were injected subcutaneously into Balb/c nude mice using $4 \times 10^6$ tumour cells per mouse ($n = 6$). Mice were observed for 1 month. The size of each subcutaneous (s.c.) tumour was measured with calipers, and the product of two dimensions of the

*© 2000 Cancer Research Campaign* 

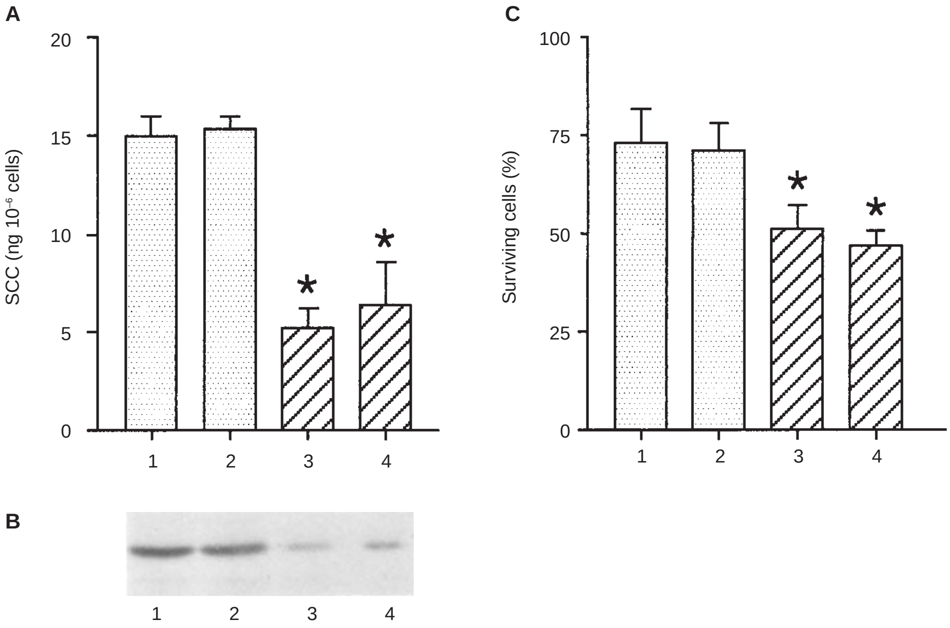

**Figure 2** Effects of the inhibition of the SCC Ag-1 expression by antisense cDNA in SKGIIIa cell line. Expression of the SCC Ag-1 was inhibited after transfection of the antisense construct. Expression level of SCC Ag-1 in each tumour cell clone was determined using the IMx system (**A**) and Western blotting (**B**). (**C**) The percentages of tumour cells surviving apoptosis after a 26 h incubation period in the presence of 10 μg $ml^{-1}$ of etoposide are shown. Lane 1, parental SKGIIIa cells; lane 2, SKGIIIa cells transduced with pCEP4 (SKGIIIa-CEP4); lanes 3 and 4, SKGIIIa cells after transduction with pCEP4-SCCAS and selection in the presence of hygromycin (SKGIIIa-AS-1 and SKGIIIa-AS-2, respectively). Asterisks indicate significant differences ($P < 0.05$) between the antisense-transduced and control cell lines tumour was defined as tumour cross-section area. Tumours were excised, sectioned and examined for the presence of apoptotic cells following staining with TUNEL reagents. The study was approved by the Institutional Animal Care and Use Committee of the University of Pittsburgh.

### Statistical analysis

Statistical analysis was done by the Student's *t*-test, Mann–Whitney's test or Wilcoxon's test, as appropriate.

### RESULTS

### Establishment of tumour cells expressing the SCC Ag-1

To be able to analyse function of the SCC Ag-1 in tumour cells, it was first necessary to establish tumour cell lines stably expressing this protein. To this end, PCI-51 cell line, which is derived from human SCC of the head and neck and does not express the SCC Ag-1, was transduced with a retroviral vector (MFG-SCC-1-neo) containing cDNA for this protein and $neo^r$ cDNA (PCI-51-SCC, PCI-51-SCCre) or with $neo^r$ cDNA alone as a control (PCI-51-NEO). To evaluate the expression level of the SCC Ag-1 in these transduced and selected cell lines, semi-quantitative RT-PCR for the SCC Ag-1 was performed. The cells transduced with *SCC Ag-1* cDNA expressed considerably higher levels of *SCC Ag-1* mRNA, in comparison to very low levels of expression in control cells, which were transduced with $neo^r$ cDNA alone (Figure 1A). The expression level of $neo^r$ message was also determined using the same method, and was found to be comparable for both groups (data not shown). Additional analyses, using Western blots and the IMx (immunoassay) method (Figure 1B), showed a large increase in the level of SCC Ag-1 protein expression in tumour cells transduced with the *SCC Ag-1* cDNA. Furthermore, the cells which were transduced repeatedly (PCI-51-SCCre) expressed more *SCC Ag-1* mRNA as well as protein compared to PCI-51-SCC. The protein levels seen in transduced cells were still within the physiological range (Numa et al, 1996). No differences in growth were observed in culture between PCI-51-SCC, PCI-51-SCCre and PCI-51-NEO cell lines (not shown).

### The SCC Ag-1 attenuates apoptosis induced by SN-38

The transduced and selected tumour cells (PCI-51-SCC, PCI-51-SCCre and PCI-51-NEO) were incubated with the anticancer drug, 7-ethyl-10-hydroxycamptothecin (SN-38), which normally induces apoptosis in tumour cells (Yoshida et al, 1993; Nakatsu et al, 1997). In fact, when these transduced cells were treated with various concentrations of SN-38, some of the cells underwent DNA fragmentation (Figure 1C). However, as shown in Figure 1D, when tumour cells expressing the SCC Ag-1 were treated with SN-38, their viability was significantly improved compared with PCI-51-NEO. Furthermore, tumour cell viability correlated with the expression level of the SCC Ag-1, as SCC cells expressing high levels of the SCC Ag-1 had significantly better viability than those expressing low levels of this protein (see Figure 1B and D).

In an attempt to more directly demonstrate the involvement of the SCC Ag-1 in the protection of tumour cells from apoptosis, antisense *SCC Ag-1* cDNA was transfected into SKGIIIa cells, which normally express SCC Ag-1 protein. As shown in Figure 2A and B, expression of the SCC Ag-1 was significantly suppressed in independently transfected tumour cell clones (SKG-AS-1 and SKG-AS-2). These clones were then incubated with apoptosis-inducing drug, etoposide (10 μg $ml^{-1}$), for 26 h. In comparison to the control clone transfected with the pCEP4 or to

 © 2000 Cancer Research Campaign

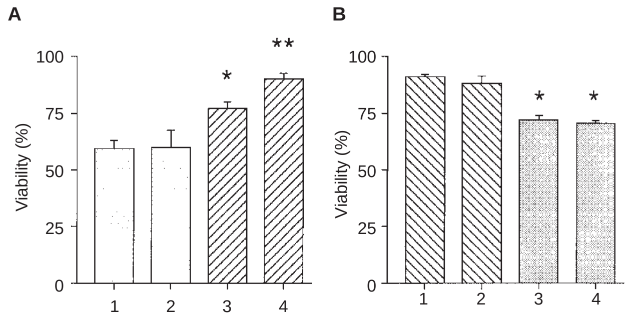

**Figure 3** Effects of the SCC Ag-1 expression on TNF-α-induced apoptosis. After preincubation of tumour targets with 10 ng ml$^{-1}$ of cycloheximide for 4 h, the cells were incubated in the presence of 10 ng ml$^{-1}$ of TNF-α and 10 ng ml$^{-1}$ of cycloheximide for the additional 24 h (**A**) or 36 h (**B**). The presence of apoptotic cells was confirmed after staining with Hoechst 33342 dye, and the percentages of viable cells in each culture was determined. (**A**) Lane 1, PCI-51; lane 2, PCI-51-NEO; lane 3, PCI-51-SCC; lane 4, PCI-51-SCCre (**B**). Lane 1, SKGIIIa; lane 2, SKGIIIa-CEP4; lane 3, SKGIIIa-AS-1; lane 4, SKGIIIa-AS-2. The data were obtained from three experiments, and means + standard deviation are shown. Significance of differences from controls (lanes 1 and 2) are indicated as follows: *$P$ < 0.05; **$P$ < 0.01. A double asterisk also indicates a significant difference ($P$ < 0.05) from lane 3 in (**A**)

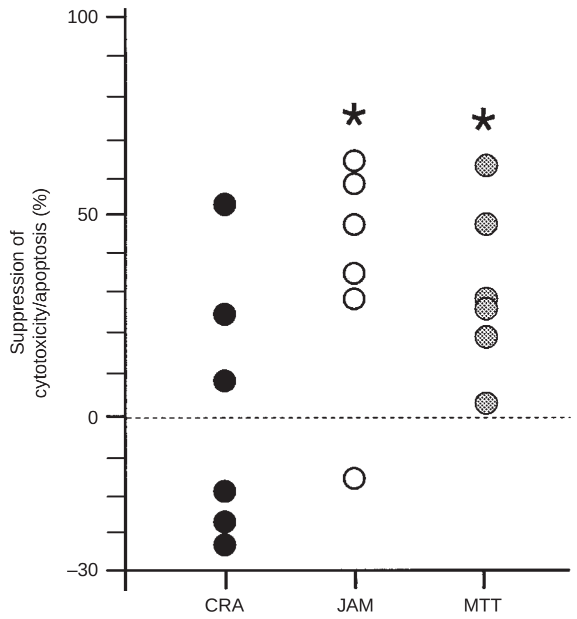

**Figure 4** Effects of the *SCC Ag-1* cDNA on the susceptibility of tumour cell targets to perforin-mediated lysis or to apoptosis mediated by A-NK cells. The data were obtained from six independent experiments, in which human A-NK cells were used as effector cells and PCI-51-SCC or PCI-51-NEO as transduced target cells. These A-NK cells were generated from different donors and had different levels of baseline activity measured with control (Neo-transduced) targets. The data are expressed as the percentage suppression of perforin-mediated lysis (CRA), apoptosis (JAM assays) or cell death (MTT assays), calculated according to the formula given in Materials and Methods. A dotted line at '0' indicates no suppression or enhancement of lysis/apoptosis in the SCC-transduced targets vs. the neo-transduced targets tested simultaneously with each A-NK cell preparation. The asterisks indicate a significant inhibition of tumour cell apoptosis detected in the JAM and MTT assays, as determined by Wilcoxon's test ($P$ < 0.05)

parental cells, tumour cells expressing low levels of the SCC Ag-1 following antisense treatment were significantly more susceptible to apoptosis induced by etoposide (Figure 2C).

### The SCC Ag-1 attenuates apoptosis induced by TNF-α

Previous experiments showed that SCC cells are susceptible to effects of exogenous TNF-α, which binds to p55 TNF-α receptor (TNFR1), inducing apoptosis (data not shown). We, therefore, wished to determine whether expression of the SCC Ag-1 in tumour cells protected them from TNF-α-induced apoptosis. First, using flow cytometry, we confirmed that surface expression of TNFR1 was not altered in PCI-51 cells after transduction with the *SCC Ag-1* cDNA (data not shown). Next, we incubated these transduced tumour cells in the presence of cycloheximide (10 ng ml$^{-1}$) and TNF-α (10 ng ml$^{-1}$) and after 24–36 h determined the level of apoptosis. As shown in Figure 3A, tumour cells transduced with the *SCC Ag-1* cDNA (PCI-51-SCC, PCI-51-SCCre) showed significantly less apoptosis ($P$ < 0.05) compared to PCI-51-Neo or parental PCI-51 cells, depending on the intracellular level of SCC Ag-1. This effect of SCC Ag-1 was confirmed using tumour cells transduced with antisense *SCC Ag-1* cDNA, which express low levels of the SCC Ag-1 (see Figure 2). The percentage of apoptotic cells of SKG IIIa-AS-1 or SKGIIIa-AS-2 tumour clones were significantly increased significantly relative to mock (pCEP4) or parental control cells (Figure 3B).

### The SCC Ag-1 attenuates apoptosis induced by A-NK cells

Inhibitory effects of the SCC Ag-1 on apoptosis in tumour cells were further analysed using A-NK cells. Co-incubation of tumour cell targets with IL-2-activated NK (A-NK) cells for 1 h was previously shown to result in apoptosis in a significant proportion of these targets (Vujanovic et al, 1995*a*). Tumour cells transduced with the *SCC Ag-1* cDNA were co-incubated with A-NK cells at different effector to target (E:T) cell ratios, and apoptosis was determined in JAM assays. The SCC Ag-1 transduced targets (PCI-51-SCC) showed significantly lower levels of apoptosis than controls transduced with the NEO gene. As shown in Figure 4, apoptosis mediated by A-NK cells, was inhibited in 5/6 experiments. The MTT assays also indicated that the expression of the SCC Ag-1 inhibited apoptosis of tumour cell targets. In contrast, the inhibitory effect of the SCC Ag-1 was not significant when $^{51}$Cr-release assays (CRA) were used to measure perforin-mediated lysis (Figure 4). Nevertheless, in 2/6 CRA, considerable suppression of lysis in the presence of the SCC Ag-1 was evident. The protective effect of the SCC Ag-1 on apoptosis in tumour targets was also confirmed by the TUNEL assay (Figure 5), which showed that significantly fewer tumour cells were undergoing DNA fragmentation in PCI-51 targets transduced with the *SCC Ag-1* cDNA than in controls.

### Mechanisms of apoptosis inhibition by the SCC Ag-1

To begin to elucidate the mechanisms involved in the observed inhibitory effects of the SCC Ag-1 on drug-, cytokine- or effector cell-induced apoptosis in tumour cells, we measured caspase-3 activity in these cells before and after their incubation with TNF-α. As shown in Figure 6, increase of caspase-3 activity was always significantly higher in control tumour cells than in tumour cells transduced with the *SCC Ag-1* cDNA. Therefore, it is likely that when present in tumour cells, the SCC Ag-1 protein acts upstream of caspase-3.

© 2000 Cancer Research Campaign 

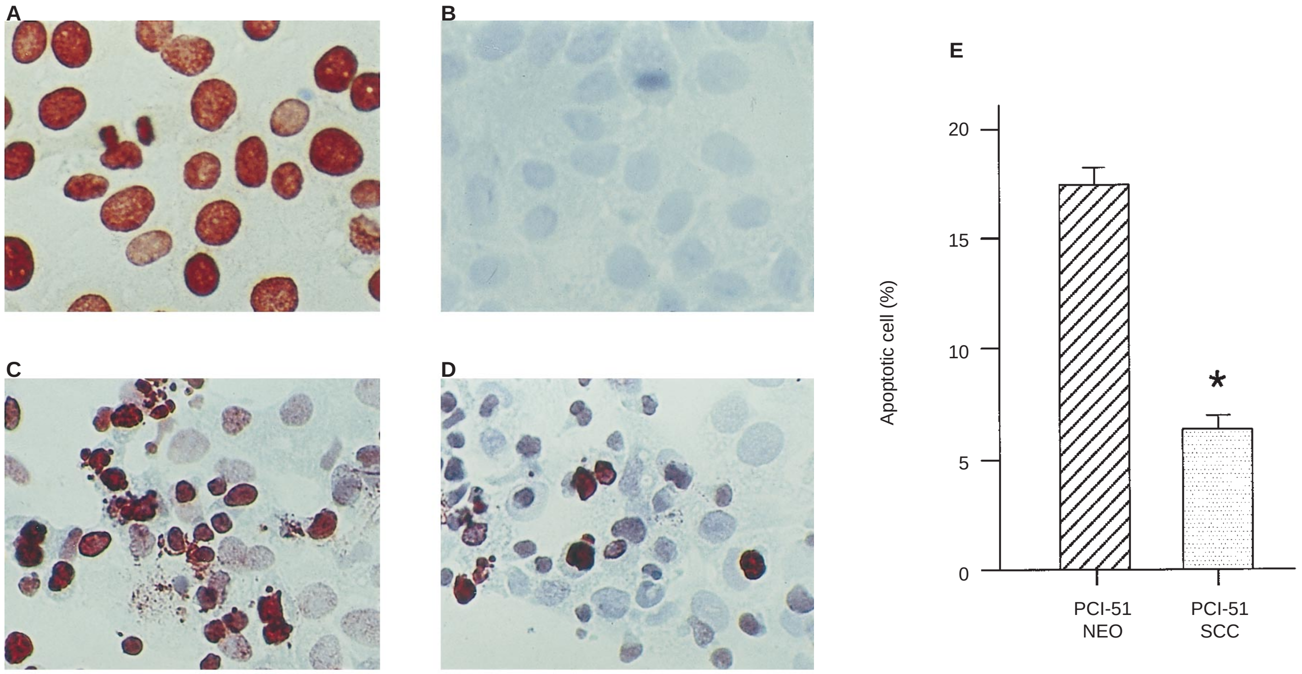

**Figure 5** Protective effects of the SCC Ag-1 on tumour cell apoptosis induced by A-NK cells, as analysed by the TUNEL method. (**A**) PCI-51-SCC treated with DNase 1 used as a positive control. (**B**) PCI-51-SCC without treatment with terminal deoxynucleotidyl transferase used as a negative control. After co-incubation with A-NK cells at the E:T ratio of 10:1, cultures of PCI-51-NEO included a considerable proportion of apoptotic cells (**C**), while cultured PCI-51-SCC cells contained fewer apoptotic cells (**D** and **E**). The apoptotic cells were counted in each of ten microscopic fields, using a high power objective (mag. × 400) and were shown as mean percentages + standard deviation in (**E**). The cells which had stained nucleus without apoptotic bodies were not counted. The asterisk indicates a significant difference ($P < 0.01$) from control

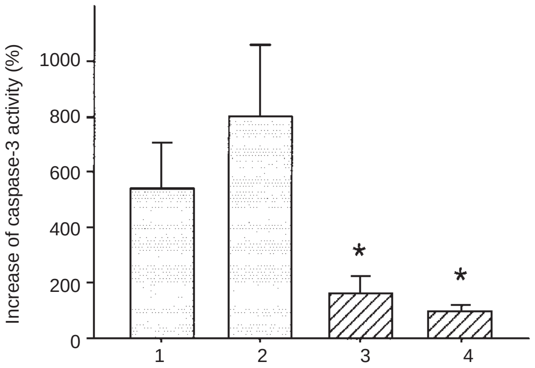

**Figure 6** Inhibition of the increase of caspase-3 activity in response to TNF-α by SCC Ag-1. After preincubation with 10 ng $ml^{-1}$ of cycloheximide for 4 h, transduced PCI-51 cells were incubated with (+) or without (–) 10 ng $ml^{-1}$ of TNF-α in the presence of 10 ng $ml^{-1}$ of cycloheximide for 15 h. The ratio of OD405 of TNF-α (+) cells and TNF-α (–) cells after incubation with the substrate was determined and fold increase in caspase-3 activity was calculated (see Methods). The results are means + standard deviation of three independent experiments. Lane 1, PCI-51; lane 2, PCI-51-NEO; lane 3, PCI-51-SCC; lane 4, PCI-51-SCCre. Significance of differences from control (lanes 1 and 2) was determined by Mann–Whitney's test and is indicated by the asterisks ($P < 0.05$)

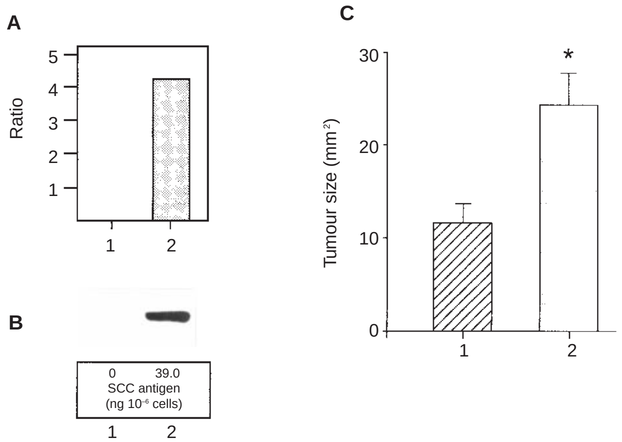

**Figure 7** In vivo growth of murine SCC cell line, KLN-205, in nude mice was transduced with *SCC Ag-1* and *neo*$^r$ cDNAs (KLN-SCC), or *neo*$^r$ cDNA alone (KLN-NEO). (**A**) Expression level of *SSC Ag-1* mRNA was determined by semi-quantitative RT-PCR (see legend to Figure 1). (**B**) The SCC Ag-1 protein was detected by Western blot analysis of cell lysates probed with anti-SCC antigen mAb (mAb-13). The level of the SCC Ag-1 in each lysate was quantitated by IMx method and is given below each lane. (**C**) The size of the tumours established by s.c. injection of $4 \times 10^6$ tumour cells was measured 1 month after injection. The data show mean tumour size + standard error of six animals studied in three experiments. Lane 1, KLN-NEO; lane 2, KLN-SCC. The asterisk indicates a significant difference in tumour size ($P < 0.05$)

### Effects of the SCC Ag-1 on tumour growth in vivo

The observation that SCC Ag-1-mediated protection of tumour targets from apoptosis in vitro prompted us to analyse this effect in vivo. To this end, nude mice were injected s.c. with KLN-SCC or KLN-NEO. The transduced KLN-SCC cells expressed the SCC Ag-1, as shown in Figure 7A and B, and the growth rate of KLN-SCC and KLN-NEO was the same in vitro (data not shown). The size of tumours induced by injection of $4 \times 10^6$ KLN-SCC cells was significantly greater than that induced by injections of control cells (KLN-NEO), as shown in Figure 7C. When the

 *© 2000 Cancer Research Campaign*

tumours induced by KLN-NEO were biopsied, sectioned and stained for TUNEL, apoptotic nuclei were detectable in a proportion of tumour cells, with fewer apoptotic nuclei seen in sections of KLN-SCC tumours (data not shown). Thus, ectopic expression of the SCC Ag-1 protected tumour cells from apoptosis in vivo and favored their survival, thereby enhancing tumour growth.

### DISCUSSION

The resistance of cancer cells to apoptosis is thought to contribute to tumour progression. One of the mechanisms responsible for resistance of tumour cells to apoptosis involves endogenous expression and activation of protease inhibitors. A number of physiologic inhibitors of apoptosis has been identified recently, including the Bcl-2 family members, FLIP, IAPs and serpins. The latter constitute a heterogenous superfamily which includes the ov-serpin family and its member, the SCC Ag-1. The biologic role of this tumour-associated protein has remained unknown, although its presence in tumours and sera of patients with SCC has been well documented (Kato et al, 1979; Mino et al, 1988; Kato, 1992). The results of our study show that ectopic expression of the SCC Ag-1 in cancer cells significantly attenuates apoptosis mediated by anti-cancer drugs, TNF-α or A-NK cells and promotes in vivo growth of the tumour.

Current data indicate that various anti-cancer drugs cause apoptosis of cancer cells and that activation of cellular proteases is involved in the drug-induced apoptotic pathway (Fisher, 1994; Ormerod et al, 1994; Desjardins and MacManus, 1995). Therefore, it is reasonable to predict that protease inhibitors might be responsible for regulation of apoptosis induced by anticancer drugs in vivo. We demonstrated that when the SCC Ag-1 was ectopically expressed in cancer cells, their apoptosis induced by SN-38 was significantly attenuated. Furthermore, inhibition of drug-induced apoptosis was related to the expression level of the SCC Ag-1 in cancer cells. Transfection of antisense *SCC Ag-1* cDNA into SCC Ag-1 positive tumour cell line (SKGIIIa) resulted in the inhibition of the SCC Ag-1 expression, and it also significantly increased susceptibility of these cells to drug-induced apoptosis. This observation is clinically important, because it illustrates that the resistance of tumour cells to anticancer drugs may be, in part, mediated by overexpression of the SCC Ag-1 in tumour tissues and that it might be prevented or decreased by the use of agents capable of blocking expression of the SCC Ag-1 gene.

Bcl-2 is a known inhibitor of apoptosis induced by various stimuli (Reed, 1997), which has also been reported to inhibit apoptosis induced by anticancer drugs (Miyashita and Reed, 1993; Ohmori et al, 1993). But expression of Bcl-2 may not be sufficient to down-regulate apoptosis in tumour cells, and Bcl-2-positive tumours were observed to have a favourable prognosis in some cases (Fontanini et al, 1995; Herod et al, 1996; Nakanishi et al, 1997). It is possible that Bcl-2 itself may be cleaved in some cells by caspases into fragments which are pro-apoptotic, as reported recently (Cheng et al, 1997). From our preliminary experiments, it appeared that function of SCC Ag-1 might not be related to that of Bcl-2 in tumour cells. Thus, overexpression of the SCC Ag-1 did not alter expression of Bcl-2 in transduced PCI-51 cells, as measured in Western blots, in comparison to that in control tumour cells (data not shown). In addition, newer evidence suggests that various pathways of apoptosis may be differentially regulated by various protease inhibitors, which target different caspases (Datta et al, 1997; Hu et al, 1998; Pan et al, 1998). In this respect, the SCC Ag-1 appears to interfere with the apoptotic pathway in tumour cells upstream from caspase-3. When the level of SCC Ag-1 expression was up-regulated by transduction of SCC Ag-1 cDNA into PCI-51 cells, an increase in caspase-3 activity was significantly smaller upon induction with TNF-α than in control cells. Further studies are in progress to determine which of the upstream caspases or proteases are targets for this serpin.

Apoptosis of cancer cells can also be induced by cytokines, such as TNF-α, or by immune killer cells, e.g. cytotoxic T lymphocytes (CTL) or NK cells. The inhibitory effect of the SCC Ag-1 on apoptosis induced by TNF-α in vitro was demonstrated in our experiments and further confirmed by inhibiting its expression with antisense *SCC Ag-1* cDNA. Our previous data indicated that exposure of tumour cells to TNF-α up-regulated expression of the SCC antigen (Numa et al, 1996). Therefore, increased expression of the SCC Ag-1 in tumour cells could represent a protective mechanism from TNF-α-induced apoptosis. IL-2-activated NK cells (A-NK cells) have been demonstrated to be able to kill targets by apoptosis (Vujanovic et al, 1993) through the TNF family receptor-ligand pairs, such as Fas/FasL or TNFR1/TNF-α (Vujanovic et al, 1995*b*) as well as by the perforin-granzyme pathway. Tumour cells transduced with the SCC Ag-1 were partially inhibited from undergoing apoptosis mediated by A-NK cells, although there was no significant effect on perforin-mediated lysis. These data suggested that the presence of the SCC Ag-1 in carcinoma cells might contribute to the defence system of tumour cells, protecting them from apoptotic death mediated by immune killer cells.

Viral serpin, CrmA (a cowpox virus protein), is a potent inhibitor of caspases 1 and 8, and it is known to inhibit apoptosis induced by TNF-α, Fas or CTL (Enari et al, 1995; Tewari and Dixit, 1995; Tewari et al, 1995*b*). Under physiologic conditions, this serpin is primarily involved in blocking apoptosis of virus-infected cells. PAI-2 which, like the SCC Ag-1, is also a member of human ov-serpin family and is coded for by the gene located on chromosome 18q21.3, has been reported to inhibit apoptosis induced by TNF-α or *Mycobacterium avium* (Dickinson et al, 1995; Gan et al, 1995). Dickinson et al suggested that the mechanism of inhibition of TNF-α-induced apoptosis by this serpin was attributable to the regulation by the host of an inflammatory process. Thus, the reported inhibitory effects of PAI-2 and CrmA on apoptosis seem to operate in the inflammatory process or infections respectively, and not in tumour growth. Several reports indicate that PAI-2 expression may be increased in certain tumours (Scherrer et al, 1991; de Vries et al, 1994). However, overexpression of PAI-2 reduces extracellular matrix degradation by urokinase-type plasminogen activator (u-PA) produced by tumour cells (Sumiyoshi et al, 1992; Nagayama et al, 1994). Thus, this process is distinct from apoptosis or its regulation. Furthermore, expression of PAI-2 appears to restrict the metastatic potential of tumour cells, resulting in a favourable prognosis (Bouchet et al, 1994; Foekens et al, 1995). In contrast, expression of the SCC Ag-1 attenuates apoptosis and promotes tumour progression. Therefore, the biologic function(s) of the SCC Ag-1 is distinct from that of CrmA and PAI-2.

Inhibition of apoptosis in tumour cells by transduction of the *SCC Ag-1* cDNA and increased susceptibility to apoptosis by transfection of the antisense cDNA, although clearly documented and significant, were not overly impressive when compared with the change in the protein level of the SCC Ag-1. This discordance could be explained by the possibility that the SCC Ag-1 might inhibit only one of several apoptotic pathways inducible by apoptotic signals. Alternately, the SCC Ag-1 may be only one of many cellular factors involved in the regulation of apoptotic pathways in tumour cells. In any event, it is highly likely that the inhibitory function(s) of the SCC Ag-1 is not restricted to a single squamous cell carcinoma cell line (PCI-51), because transduction of SCC Ag-1 cDNA to non-squamous cell line (e.g. K562) gave analogous results (data not shown).

In summary, our results suggest that the SCC Ag-1, a member of the human serpin family and a tumour-associated protein, is functionally linked to the apoptotic pathway(s) in squamous cell carcinoma and other tumour cells. The expression level of the SCC Ag-1 in cancer cells appears to be, in part, responsible for resistance of these cells to apoptosis in vitro and to tumour progression and growth in vivo.

### ACKNOWLEDGEMENTS

We thank Drs Kazuyuki Hatakeyama, Tadamichi Suzuki, Hideaki Tahara, Torsten E Reichert and Ming-Whun Sung for advice and helpful comments received in the course of these studies. These studies were supported in part by the NIH grant P0-1 DE 12321 to Theresa L Whiteside, PhD.

### REFERENCES

Alnemri ES, Livingston DJ, Nicholson DW, Salvesen G, Thornberry NA, Wong WW and Yuan J (1996) Human ICE/CED-3 protease nomenclature [letter]. *Cell* **87**: 171

Belin D, Wohlwend A, Schleuning WD, Kruithof EK and Vassalli JD (1989) Facultative polypeptide translocation allows a single mRNA to encode the secreted and cytosolic forms of plasminogen activators inhibitor 2. *EMBO J* **8**: 3287–3294

Bouchet C, Spyratos F, Martin PM, Hacene K, Gentile A and Oglobine J (1994) Prognostic value of urokinase-type plasminogen activator (uPA) and plasminogen activator inhibitors PAI-1 and PAI-2 in breast carcinomas. *Br J Cancer* **69**: 398–405

Cheng EH, Kirsch DG, Clem RJ, Ravi R, Kastan MB, Bedi A, Ueno K and Hardwick JM (1997) Conversion of Bcl-2 to a Bax-like death effector by caspases. *Science* **278**: 1966–1968

Danos O and Mulligan RC (1988) Safe and efficient generation of recombinant retroviruses with amphotropic and ecotropic host ranges. *Proc Natl Acad Sci USA* **85**: 6460–6464

Datta R, Kojima H, Banach D, Bump NJ, Talanian RV, Alnemri ES, Weichselbaum RR, Wong WW and Kufe DW (1997) Activation of a CrmA-insensitive, p35-sensitive pathway in ionizing radiation-induced apoptosis. *J Biol Chem* **272**: 1965–1969

de Vries TJ, Quax PH, Denijn M, Verrijp KN, Verheijen JH, Verspaget HW, Weidle UH, Ruiter DJ and van Muijen GN (1994) Plasminogen activators, their inhibitors, and urokinase receptor emerge in late stages of melanocytic tumour progression. *Am J Pathol* **144**: 70–81

Desjardins LM and MacManus JP (1995) An adherent cell model to study different stages of apoptosis. *Exp Cell Res* **216**: 380–387

Dickinson JL, Bates EJ, Ferrante A and Antalis TM (1995) Plasminogen activator inhibitor type 2 inhibits tumour necrosis factor alpha-induced apoptosis. Evidence for an alternate biological function. *J Biol Chem* **270**: 27894–27904

Duk JM, Groenier KH, de Bruijn HW, Hollema H, ten Hoor KA, van der Zee AG and Aalders JG (1996) Pretreatment serum squamous cell carcinoma antigen: a newly identified prognostic factor in early-stage cervical carcinoma. *J Clin Oncol* **14**: 111–118

Enari M, Hug H and Nagata S (1995) Involvement of an ICE-like protease in Fas-mediated apoptosis. *Nature* **375**: 78–81

Fisher DE (1994) Apoptosis in cancer therapy: crossing the threshold. [Review]. *Cell* **78**: 539–542

Foekens JA, Buessecker F, Peters HA, Krainick U, van Putten WL, Look MP, Klijn JG and Kramer MD (1995) Plasminogen activator inhibitor-2: prognostic relevance in 1012 patients with primary breast cancer. *Cancer Res* **55**: 1423–1427

Fontanini G, Vignati S, Bigini D, Mussi A, Lucchi M, Angeletti CA, Basolo F and Bevilacqua G (1995) Bcl-2 protein: a prognostic factor inversely correlated to p53 in non-small-cell lung cancer. *Br J Cancer* **71**: 1003–1007

Gan H, Newman GW and Remold HG (1995) Plasminogen activator inhibitor type 2 prevents programmed cell death of human macrophages infected with *Mycobacterium avium*, serovar 4. *J Immunol* **155**: 1304–1315

Herod JJ, Eliopoulos AG, Warwick J, Niedobitek G, Young LS and Kerr DJ (1996) The prognostic significance of Bcl-2 and p53 expression in ovarian carcinoma. *Cancer Res* **56**: 2178–2184

Hirabayashi H, Yasumura S, Lin WC, Amoscato A, Johnson JT, Herberman RB and Whiteside TL (1995) Production by human squamous cell carcinoma of a factor inducing activation and proliferation of immune cells. *Arch Otolaryngol Head Neck Surg* **121**: 285–292

Hu Y, Benedict MA, Wu D, Inohara N and Nunez G (1998) Bcl-XL interacts with Apaf-1 and inhibits Apaf-1-dependent caspase-9 activation. *Proc Natl Acad Sci USA* **95**: 4386–4391

Kato H (1992) Squamous cell carcinoma antigen. In: *Serological Cancer Markers*, Sell S (ed), pp. 437–451. Humana Press: Clifton

Kato H and Torigoe T (1977) Radioimmunoassay for tumour antigen of human cervical squamous cell carcinoma. *Cancer* **40**: 1621–1628

Kato H, Miyauchi F, Morioka H, Fujino T and Torigoe T (1979) Tumor antigen of human cervical squamous cell carcinoma: correlation of circulating levels with disease progress. *Cancer* **43**: 585–590

Kato H, Nagaya T and Torigoe T (1984) Heterogeneity of a tumour antigen TA-4 of squamous cell carcinoma in relation to its appearance in the circulation. *Gann* **75**: 433–435

Mino N, Iio A and Hamamoto K (1988) Availability of tumour-antigen 4 as a marker of squamous cell carcinoma of the lung and other organs. *Cancer* **62**: 730–734

Miyashita T and Reed JC (1993) Bcl-2 oncoprotein blocks chemotherapy-induced apoptosis in a human leukemia cell line. *Blood* **81**: 151–157

Nagashima S, Kashii Y, Reichert TE, Suminami Y, Suzuki T and Whiteside TL (1997) Human gastric carcinoma transduced with the IL-2 gene: increased sensitivity to immune effector cells in vitro and in vivo. *Int J Cancer* **72**: 174–183

Nagayama M, Sato A, Hayakawa H, Urano T, Takada Y and Takada A (1994) Plasminogen activators and their inhibitors in non-small cell lung cancer. Low content of type 2 plasminogen activator inhibitor associated with tumour dissemination. *Cancer* **73**: 1398–1405

Nakanishi H, Ohsawa M, Naka N, Uchida A, Ochi T and Aozasa K (1997) Immunohistochemical detection of bcl-2 and p53 proteins and apoptosis in soft tissue sarcoma: their correlations with prognosis. *Oncology* **54**: 238–244

Nakatsu S, Kondo S, Kondo Y, Yin D, Peterson JW, Kaakaji R, Morimura T, Kikuchi H, Takeuchi J and Barnett GH (1997) Induction of apoptosis in multidrug resistant (MDR) human glioblastoma cells by SN-38, a metabolite of the camptothecin derivative CPT-11. *Cancer Chemother Pharmacol* **39**: 417–423

Nawata S, Tsunaga N, Numa F, Tanaka T, Nakamura K and Kato H (1995) Serine protease inhibitor activity of recombinant squamous cell carcinoma antigen towards chymotrypsin, as demonstrated by sodium dodecyl sulfate-polyacrylamide gel electrophoresis. *Electrophoresis* **16**: 1027–1030

Nawata S, Nakamura K, Tanaka T, Numa F, Suminami Y, Tsunaga N, Kakegawa H, Katunuma N and Kato H (1997) Electrophoretic analysis of the ‘cross-class’ interaction between novel inhibitory serpin, squamous cell carcinoma antigen-1 and cysteine proteinases. *Electrophoresis* **18**: 784–789

Numa F, Takeda O, Nakata M, Nawata S, Tsunaga N, Hirabayashi K, Suminami Y, Kato H and Hamanaka S (1996) Tumour necrosis factor-α stimulates the production of pquamous cell carcinoma antigen in normal squamous cells. *Tumour Biol* **17**: 97–101

Ohashi T, Boggs S, Robbins P, Bahnson A, Patrene K, Wei FS, Wei JF, Li J, Lucht L, Fei Y & et al (1992) Efficient transfer and sustained high expression of the human glucocerebrosidase gene in mice and their functional macrophages following transplantation of bone marrow transduced by a retroviral vector. *Proc Natl Acad Sci USA* **89**: 11332–11336

Ohmori T, Podack ER, Nishio K, Takahashi M, Miyahara Y, Takeda Y, Kubota N, Funayama Y, Ogasawara H, Ohira T & et al (1993) Apoptosis of lung cancer cells caused by some anti-cancer agents (MMC, CPT-11, ADM) is inhibited by bcl-2. *Biochem Biophys Res Commun* **192**: 30–36

Ormerod MG, O’Neill CF, Robertson D and Harrap KR (1994) Cisplatin induces apoptosis in a human ovarian carcinoma cell line without concomitant internucleosomal degradation of DNA. *Exp Cell Res* **211**: 231–237

Pan G, O’Rourke K and Dixit VM (1998) Caspase-9, Bcl-XL, and Apaf-1 form a ternary complex. *J Biol Chem* **273**: 5841–5845

Potempa J, Korzus E and Travis J (1994) The serpin superfamily of proteinase inhibitors: structure, function, and regulation. [Review]. *J Biol Chem* **269**: 15957–15960

 © 2000 Cancer Research Campaign

Pross HF, Baines MG, Rubin P, Shragge P and Patterson MS (1981) Spontaneous human lymphocyte-mediated cytotoxicity against tumour target cells. IX. The quantitation of natural killer cell activity. *J Clin Immunol* **1**: 51–63

Reed JC (1995) Regulation of apoptosis by bcl-2 family proteins and its role in cancer and chemoresistance. [Review]. *Curr Opin Oncol* **7**: 541–546

Reed JC (1997) Double identity for proteins of the Bcl-2 family. [Review]. *Nature* **387**: 773–776

Scherrer A, Kruithof EK and Grob JP (1991) Plasminogen activator inhibitor-2 in patients with monocytic leukemia. *Leukemia* **5**: 479–486

Schneider SS, Schick C, Fish KE, Miller E, Pena JC, Treter SD, Hui SM and Silverman GA (1995) A serine proteinase inhibitor locus at 18q21.3 contains a tandem duplication of the human squamous cell carcinoma antigen gene. *Proc Natl Acad Sci USA* **92**: 3147–3151

Suminami Y, Kishi F, Sekiguchi K and Kato H (1991) Squamous cell carcinoma antigen is a new member of the serine protease inhibitors. *Biochem Biophys Res Commun* **181**: 51–58

Sumiyoshi K, Serizawa K, Urano T, Takada Y, Takada A and Baba S (1992) Plasminogen activator system in human breast cancer. *Int J Cancer* **50**: 345–348

Takeda A, Yamamoto T, Nakamura Y, Takahashi T and Hibino T (1995) Squamous cell carcinoma antigen is a potent inhibitor of cysteine proteinase cathepsin L. *FEBS Lett* **359**: 78–80

Takeshima N, Nakamura K, Takeda O, Morioka, H, Tamura, H, Takasugi N and Kato H (1990) Individualization of the cutoff value for serum squamous-cell carcinoma antigen using a sensitive enzyme immunoassay. *Tumour Biol* **11**: 167–172

Tewari M and Dixit VM (1995) Fas- and tumor necrosis factor-induced apoptosis is inhibited by the poxvirus crmA gene product. *J Biol Chem* **270**: 3255–3260

Tewari M, Quan LT, O'Rourke K, Desnoyers S, Zeng Z, Beidler DR, Poirier GG, Salvesen GS and Dixit VM (1995*a*) Yama/CPP32 beta, a mammalian homolog of CED-3, is a CrmA-inhibitable protease that cleaves the death substrate poly (ADP-ribose) polymerase. *Cell* **81**: 801–809

Tewari M, Telford WG, Miller RA and Dixit VM (1995*b*) CrmA, a poxvirus-encoded serpin, inhibits cytotoxic T-lymphocyte-mediated apoptosis. *J Biol Chem* **270**: 22705–22708

Vujanovic NL, Rabinowich H, Lee YJ, Jost L, Herberman RB and Whiteside TL (1993) Distinct phenotypic and functional characteristics of human natural killer cells obtained by rapid interleukin 2-induced adherence to plastic. *Cell Immunol* **151**: 133–157

Vujanovic NL, Nagashima S, Giorda R, Herberman RB and Whiteside TL (1995*a*) Multiple mechanisms of killing of tumour cell targets by IL-2-activated natural killer cells. In: *Abstract Book of the 9th International Congress of Immunology (San Francisco, CA*), pp. 486(2883)

Vujanovic NL, Yasumura S, Hirabayashi H, Lin WC, Watkins S, Herberman RB and Whiteside TL (1995*b*) Antitumour activities of subsets of human IL-2-activated natural killer cells in solid tissues. *J Immunol* **154**: 281–289

Vujanovic NL, Nagashima S, Herberman RB and Whiteside TL (1996) Non-secretory apoptotic killing by human NK cells. *J Immunol* **157**: 1117–1126

Yoshida A, Ueda T, Wano Y and Nakamura T (1993) DNA damage and cell killing by camptothecin and its derivative in human leukemia HL-60 cells. *Jpn J Cancer Res* **84**: 566–573

Zitvogel L, Tahara H, Cai Q, Storkus WJ, Muller G, Wolf SF, Gately M, Robbins PD and Lotze MT (1994) Construction and characterization of retroviral vectors expressing biologically active human interleukin-12. *Hum Gene Ther* **5**: 1493–1506

*© 2000 Cancer Research Campaign* 
